# On the journey from nematode to human, scientists dive by the zebrafish cell lineage tree

**DOI:** 10.1186/s13059-018-1453-x

**Published:** 2018-05-29

**Authors:** Ehud Shapiro

**Affiliations:** Department of Computer Science and Applied Math, Weizmann Institute of Science, Rehovot, Israel

## Abstract

Three recent single-cell papers use novel CRISPR-Cas9-sgRNA genome editing methods to shed light on the zebrafish cell lineage tree.

## Whole-organism cell lineage tree

The cell lineage tree of the nematode *Caenorhabditis elegans* was uncovered four decades ago by painstaking observation of the nematode’s development. The tree, typically drawn upside-down, has the root at the top, representing the fertilized zygote, the leaves at the bottom, representing the organism’s extant cells, and internal branches representing past cells that have divided. Cell lineage trees are typically labeled with the types of the cells and, in the case of the small (959 somatic cells) and deterministic cell lineage tree of *C. elegans*, also with a unique identifier for each cell. This Nobel-winning work has been the bedrock of ample research on *C. elegans* biology ever since.

Unfortunately, science has yet to know the cell lineage tree of a more complex model organism. Mathematically, naturally occurring somatic mutations induced during normal cell division carry enough information to specify with high precision the organismal cell lineage trees of complex organisms, such as mouse and possibly even human [1]. Utilizing phylogenetic analyses of naturally occurring somatic mutations for the discovery of cell lineage trees faces two major limitations at present. First, eliciting this mutational information, such as by high-coverage single-cell whole-genome sequencing of every cell of a complex organism, is prohibitive with today’s technologies. Second, such a tree, obtained solely from retrospective phylogenetic analysis of the organism’s extant cells, would be blank, with no further information on the nature of the cells, and hence rather uninformative. To label the tree with cell types, transcriptomic (or other) analysis of each cell is needed in addition to its genomic analysis. While single-cell transcriptomics is progressing in leaps and bounds and is now the cornerstone technology of the international Human Cell Atlas project, integrated single-cell genome and transcriptome analysis is still in its infancy [2].

Fortunately, a new idea has recently emerged. It is possible to use CRISPR-Cas9-sgRNA genome editing to address these two problems simultaneously. In accordance with the multiple discovery theory, the idea is presented in three independent, almost simultaneous, publications, all applying it to the discovery of the zebrafish cell lineage tree [3–5].

Uncovering zebrafish cell lineages by scarring its genome, waiting, then fishing the scars, the method uses CRISPR-Cas9 to inflict random edits to the cell’s genome, called genomic scars, at specifically chosen subgenomic (sgRNA)-guided locations. Such scars are, in fact, induced somatic mutations heritable via cell division and can be used, with the help of phylogenetic analysis tools, to reconstruct lineage relationships among the organism’s scarred cells. As the putative locations of these scars within the genome are known, they can be recovered by targeted sequencing, eschewing the need for high-coverage single-cell whole-genome sequencing. To eliminate the need for simultaneous genomic and transcriptomic analysis of individual cells, these scars are inflicted in expressed genomic loci. Thus, single-cell RNA sequencing can recover both a cell’s type and its expressed genomic scars. To ensure the scars do not affect organism development, they are applied only to a nonfunctional transgene such as GFP, which is incorporated in a sufficient number of copies in the genome to support ample scarring. Three variations of this combined concept, termed ScarTrace [3], scGESTALT [5], and LINNAEUS [4], have been applied by the three teams to analyze various aspects of the zebrafish cell lineage tree, focusing on early development [4], the brain [5] and the entire organism, with focus on the immune system and eye [3]. Highlights of their research findings include showing that a subpopulation of resident macrophages in the fin has a different origin than monocytes in the marrow [3]; that erythrocytes generated by primitive hematopoiesis have a distinct origin from those generated by definitive hematopoiesis [4]; and that the heart harbors two seemingly very similar endocardial/endothelial cell types which have very different origins [4].

## Diving deeper into the zebrafish cell lineage tree

The research milestone reached by these three papers is worth celebrating, as it offers a completely new way to peer into complex organism development. Yet, it is a small step in a long journey. Even within the realm of zebrafish, many limitations have yet to be overcome.

First, the number of cells analyzed by these papers is measured in the tens of thousands, a far cry from the adult zebrafish estimated 100,000,000 cells. Significant scaling of the method in all dimensions, as well as drastic declines in sequencing costs, is needed to reconstruct the full zebrafish cell lineage tree.

Second, unlike natural somatic mutations, which occur continuously during normal cell division, the methods described inflicted CRISPR-Cas9 scarring only once or twice during the organism’s lifespan. Continuous scarring is needed for full cell lineage tree reconstruction.

Third, while phylogenetic analysis tools have been improving for decades, phylogenetic cell lineage reconstruction has specific needs, notably coping with noisy, partial, or missing single-cell genomic data, and reconstructing ever-increasing lineage trees, orders of magnitude larger than what has been previously attempted. Novel and better algorithms have to be developed to cope with these challenges.

Fourth, while cell type and lineage are useful information, without cell location the resulting picture would still be rather partial. Methods for in situ RNA sequencing which could incorporate genome scarring to uncover simultaneously cell location, cell type, and cell lineage would give a more complete picture of organism development.

Fifth, while the number branches between a cell and the root measures the number of cell divisions it underwent since the zygote, it does not measure time. There could be parts of the tree that extend slowly throughout the adult life and parts that progress quickly during early life then stop. The timing of cell division, differentiation, and renewal is a major question of fundamental biological importance. While the timestamps of the root and leaves of an organismal cell lineage tree are determined by the actual experiment that generated it, timestamps of internal nodes can only be inferred retrospectively, like type and location information, with the aid of yet-unavailable mathematical methods applied to snapshots taken at different time points.

Sixth, a fundamental limitation of any retrospective method, including this one, is that it cannot peer into the past, only speculate about it. Specifically, single-cell RNA-sequencing can provide information only on extant cells, namely the leaves of the cell lineage tree. Any knowledge on past internal tree nodes can only be inferred. Conversely, analysis of an organism at cellular resolution using current methods requires its sacrifice, obviously preventing further organism development, so peering into its future is also impossible. If organism development is deterministic, as in *C. elegans*, internal nodes can be analyzed by freezing development of individuals at different time points for analysis, and then coalescing the resulting partial lineage trees into a unified lineage tree. However, complex organisms may not be deterministic, in which case simple coalescence of cell lineage trees, even of clones, might not be possible. Snapshots at cellular resolution of different individual organisms at different stages of development would be needed and helpful of course, but they cannot be simply coalesced. Yet-unavailable mathematical and computational methods have to be developed to make sound inferences of the type and location of internal nodes from information on the cells at the leaves of a cell lineage tree of a complex organism.

## From zebrafish to mouse and—ultimately—to the human cell lineage tree

Climbing up the model organism hierarchy, the mouse is an obvious next target of this method, as a lot of cell lineage knowledge exists as a backdrop to verify the method, as well as to improve upon. The mouse can also be a stepping stone for human cell lineage reconstruction. A key hurdle for any human cell lineage reconstruction method is lack of a ground truth to measure against. While a cell lineage tree can be easily scribbled, verifying its relationship to the actual developmental history of an organism is far from trivial. If and when genome scarring proves a reliable method for mouse cell lineage reconstruction, it can serve as a ground truth for testing, in mouse, retrospective cell lineage reconstruction using naturally occurring somatic mutations. Due to ethics considerations, this may be the only viable method for uncovering the human cell lineage tree.

To conclude, let’s ask: why bother? What will we gain at the end of this journey, if we know the human cell lineage tree? The answers are nothing short of dramatic. I can fairly say that truthful human cell lineage trees, fully labeled with type, temporal, and spatial information, would provide long-sought answers to the most profound open questions in human biology and medicine. Here are three examples: First, the human cell lineage tree can summarize the answers to all open questions on human development, at cellular, if not molecular, resolution. Second, such a tree would end the fierce controversies regarding regeneration during adulthood, which rage in every human-organ research community I know. For example, do beta cells renew [6]? The heart [7]? Neurons [8, 9]? Oocytes [10]? The answers will be found in the human cell lineage tree. Third, it would also be able to explain disease dynamics and answer questions such as: where do metastases come from? Which cells initiate relapse after treatment? The answers lie in the patients’ cell lineage trees [2].

Obtaining knowledge of the human cell lineage tree in development, aging, and disease on par with our current knowledge of the human genome will take decades. But this is a journey worth taking, and a journey science must take.
